# Single nucleotide polymorphisms concordant with the horned/polled trait in Holsteins

**DOI:** 10.1186/1756-0500-1-128

**Published:** 2008-12-08

**Authors:** Edward J Cargill, Nick J Nissing, Michael D Grosz

**Affiliations:** 1 Monsanto Company, 800 N. Lindbergh Blvd, St Louis, MO 63167, USA

## Abstract

**Background:**

Cattle that naturally do not grow horns are referred to as polled, a trait inherited in a dominant Mendelian fashion. Previous studies have localized the polled mutation (which is unknown) to the proximal end of bovine chromosome 1 in a region approximately 3 Mb in size. While a polled genetic test, Tru-Polled™, is commercially available from MetaMorphix Inc., Holsteins are not a validated breed for this test.

**Findings:**

Approximately 160 kb were sequenced within the known polled region from 12 polled and 12 horned Holsteins. Analysis of the polymorphisms identified 13 novel single nucleotide polymorphisms (SNPs) that are concordant with the horned/polled trait. Three of the 13 SNPs are located in gene coding or regulatory regions (*e.g*., the untranslated region, or UTR) where one is located in the 3'UTR of a gene and the other two are located in the 5'UTR and coding region (synonymous SNP) of another gene. The 3'UTR of genes have been shown to be targets of microRNAs regulating gene expression. *In silico *analysis indicates the 3'UTR SNP may disrupt a microRNA target site.

**Conclusion:**

These 13 novel SNPs concordant with the horned/polled trait in Holsteins represent a test panel for the breed and this is the first report to the authors' knowledge of SNPs within gene coding or regulatory regions concordant with the horned/polled trait in cattle. These SNPs will require further testing for verification and further study to determine if the 3'UTR SNP may have a functional effect on the polled trait in Holsteins.

## Background

Cattle that naturally do not grow horns are termed polled, a trait inherited in an autosomal dominant fashion [1,2]. De-horning is a common practice in the cattle industry as the presence of horns can lead to injuries such as bruised carcasses and hence, economic loss. Polled cattle are desirable; however, the frequency of the trait is minimal due to the management practice of de-horning calves, which prohibits the later selection and breeding of naturally polled individuals. While de-horning is a management solution, the issue ranks as a high concern with producers and packers [3,4]. In addition, the process of de-horning creates stress for the cattle [5] and may be viewed as inhumane.

While a polled genetic test is commercially available from MetaMorphix Inc., Holsteins are not listed as a validated breed for the Tru-Polled™ test [6]. The breeds the Tru-Polled™ test is validated for are Charolais, Gelbvieh, Hereford, Limousin, Salers, and Simmental [6]. Creation of a polled genetic test for Holsteins, the major dairy breed, would be valuable to the dairy industry for inclusion of this trait in selection programs utilizing genetic markers.

The polled mutation in *Bos taurus*, which is unknown, was localized to the proximal end of bovine chromosome 1 (BTA01) with microsatellite markers [7]. More recent efforts to fine-map the polled locus have included additional microsatellite marker and gene mapping [8-11] and the creation of a BAC-based physical map of the polled region [12]. The location of the most proximal gene, *ATP5O*, and most distal gene, *KRTAP8*, of the polled region from these cited sources corresponds to approximately 0.6 Mb and 3.9 Mb respectively on the public bovine genome assembly version 4.0 [13]. One study [11] did fine map the polled region to a 1 Mb segment that corresponds to approximately 0.6 Mb to 1.6 Mb from the proximal end of BTA01.

**Figure 1:**
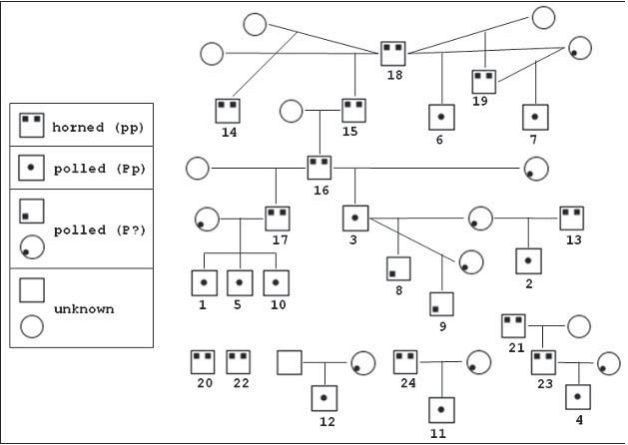
Pedigree illustration of the Holstein panel, where ID numbers indicate the horned or polled Holstein bulls included in the panel.

The objective of this work was to identify single nucleotide polymorphisms (SNPs) associated with the polled trait in Holsteins by sequencing targeted regions of the proximal end of BTA01 on a panel of 12 polled and 12 horned bulls (Figure 1). Polymorphisms found to be associated with the polled trait and located in genes, specifically coding and regulatory regions (*e.g*., untranslated region, or UTR) will be analyzed *in silico *to determine if there is any potential functional effect.

## Results

### Polymorphism detection

Approximately 160 kb were sequenced from the 0.6 Mb to 3.9 Mb polled region on BTA01 for polymorphism detection by targeting known gene coding and regulatory regions as well as putative regulatory regions (Table 1). Putative regulatory elements were identified by scanning gene introns and inter-genic sequence regions with WWW Promoter Scan [14]. Overall 261 polymorphisms, including SNPs and insertion-deletion polymorphisms (INDELs), were characterized within and around the targeted genes and putative regulatory elements in the polled region. Only SNPs concordant with the polled trait are described in this report and none of the INDELs were concordant with the trait.

**Table 1:** Genes targeted for polymorphism detection within the polled region on BTA01.^1^

**Gene**	**Description**	**NCBI GeneID**
ITSN1	intersectin 1	510879

IFNAR2	interferon (alpha, beta and omega) receptor 2	282258

OLIG1	oligodendrocyte transcription factor 1	539307

OLIG2	oligodendrocyte transcription factor 2	539695

LOC784884	hypothetical protein LOC784884	784884

SYNJ1	synaptojanin 1	282087

C21orf59	chromosome 21 open reading frame 59	535482

C21orf63	chromosome 21 open reading frame 63	516536

MRAP	melanocortin 2 receptor accessory protein	505743

SOD1	superoxide dismutase 1, soluble	281495

KRTAP11	keratin associated protein 11-1	781604

KRTAP8	keratin associated protein 8-1	782239

^1 ^Bovine specific NCBI (National Center for Biotechnology Information) GeneIDs [19] can be used to retrieve all public information regarding the genes. The gene sequences were retrieved from the public bovine genome sequence assembly, which was version 3.1 at the time [13].

### Polymorphisms concordant with the polled trait

Of the 261 polymorphisms identified in the polled region on BTA01, 13 SNPs were found to be concordant with the polled trait in the Holstein panel. Concordance is defined as all 12 horned bulls being homozygous for one polymorphism allele and all 12 polled bulls being heterozygous or homozygous for the opposite polymorphism allele. The 13 SNPs concordant with the polled trait are listed in Table 2. Multiple polymorphisms were also identified next to and between these 13 SNPs, but only the SNPs listed in Table 2 were 100% concordant (as defined above and shown in Table 3 for SNP bSYNJ1_C3981T) with the polled trait in the Holstein panel. Primers to amplify PCR products containing the 13 SNPs are listed in Table 4.

**Table 2:** SNPs concordant with the polled trait in the Holstein panel.^1^

**Gene**	**SNP Name**	**SNP Alleles**	**SNP Type**	**NCBI dbSNP**
*IFNAR2*	bCLCVS1_TC_238	C (polled) T (horned)	intron	ss105143724

*SYNJ1*	bSYNJ1_CT_271	T (polled) C (horned)	intron	ss105143725

*SYNJ1*	bSYNJ1_CT_131	T (polled) C (horned)	intron	ss105143726

*SYNJ1*	bSYNJ1_AG_244	G (polled) A (horned)	intron	ss105143727

*SYNJ1*	bSYNJ1_C3981T	T (polled) C (horned)	3'UTR	ss105143728

n/a	bCLF1O1_GA_193	A (polled) G (horned)	inter-genic	ss105143729

*C21orf59*	bC2159_C-193T	T (polled) C (horned)	5'UTR	ss105143730

*C21orf59*	bC2159_T372C	C (polled) T (horned)	synonymous	ss105143731

*C21orf63*	bC2163_AG_454	G (polled) A (horned)	intron	ss105143732

*C21orf63*	bC2163_GA_333	A (polled) G (horned)	intron	ss105143733

*C21orf63*	bC2163_GA_252	G (polled) A (horned)	intron	ss105143734

*C21orf63*	bC2163_AC_126	A (polled) C (horned)	intron	ss105143735

*C21orf63*	bC2163_CT_145	C (polled) T (horned)	intron	ss105143736

^1^SNP alleles concordant with the polled trait are listed. SNP Type is the location within a gene (intron, 5' or 3'UTR, synonymous = no amino acid change) or between genes (inter-genic).

**Table 3:** Genotypes of the 24 bulls in the Holstein panel for SNP bSYNJ1_C3981T.^1^

**Holstein**	**Horned/Polled**	**bSYNJ1_C3981T**	**Holstein**	**Horned/Polled**	**bSYNJ1_C3981T**
1	polled	CT	13	horned	CC

2	polled	CT	14	horned	CC

3	polled	CT	15	horned	CC

4	polled	CT	16	horned	CC

5	polled	CT	17	horned	CC

6	polled	CT	18	horned	CC

7	polled	CT	19	horned	CC

8	polled	CT	20	horned	CC

9	polled	CT	21	horned	CC

10	polled	CT	22	horned	CC

11	polled	CT	23	horned	CC

12	polled	CT	24	horned	CC

^1 ^Holstein numbers and horned/polled status correspond to Figure 1; bSYNJ1_C3981T genotypes listed as detected by sequencing as described in Materials and Methods.

**Table 4:** PCR primers, forward (F) and reverse (R) listed 5' to 3', PCR product size in base pairs, and base pair position within the PCR product for the 13 SNPs concordant with the polled trait in Holsteins.

**SNP Name**	**Primer Set**	**PCR Size**	**Position**
bC2163_AG_454	F: CTTGCCCTTGACTTTCTCTC	501	454
			
	R: CTCTCCTCCTCCTCAGTTTG		

bC2163_GA_333	F: GATTCACCACCACACTGGA	740	333
			
	R: CACCATCACAAAGCAGAAAA		

bC2163_GA_252	F: GGCAATGTCACCATCAACC	383	252
			
	R: CAAAGAACAGAAAGCCAACAAG		

bC2163_AC_126	F: TCTAAGTGCCTGTAATCTGTGAG	563	126
			
	R: AGGTCTTTTGCGGTGTAATC		

bC2163_CT_145	F: TCTAAGTGCCTGTAATCTGTGAG	563	145
			
	R: AGGTCTTTTGCGGTGTAATC		

bCLCVS1_TC_238	F: CTAAGGAATTTGACTCTCACCTCT	567	238
			
	R: CTACTCCCCTTCTGCTACCC		

bCLF1O1_GA_193	F: CTGGTCGGTTTGAGGGTTG	559	193
			
	R: AGCAGGAACTGGCTCTCGT		

bC2159_C-193T	F: CTGGTCGGTTTGAGGGTTG	559	320
			
	R: AGCAGGAACTGGCTCTCGT		

bC2159_T372C	F: TACTTTCTCTCATCTCACTTCCTC	370	165
			
	R: GTTTGTTTCCTTTGCCTCTG		

bSYNJ1_CT_271	F: TCACTGGATGTATGTCTGTTGG	461	271
			
	R: TCAGGAAGAGTAAATGGGTTTC		

bSYNJ1_CT_131	F: TCAGTATTTGTGGCATGTGG	508	131
			
	R: CTCGACTTGGTCTCACTGG		

bSYNJ1_AG_244	F: GAATGACAAAGAGGCAGAGG	497	244
			
	R: AGAGCTGGCCCTAAAGAAAG		

bSYNJ1_C3981T	F: AACCACCAGAGTAACAGACTACAC	471	190
			
	R: CTGTCGGTGAAAGGATTTG		

Of the 13 SNPs identified as concordant with the polled trait (Table 2), only three are located in a gene's coding or regulatory (*i.e*., promoter or UTR) region. The other 10 SNPs are located in introns or are inter-genic and not present in a putative regulatory element as analyzed by WWW Promoter Scan [14]. The bSYNJ1_C3981T polymorphism is located in the 3'UTR of the *SYNJ1 *gene, and SNPs bC2159_C-193T and bC2159_T372C are located in the 5'UTR and coding region of the *C21orf59 *gene, respectively. The bC2159_T372C polymorphism is a synonymous SNP, with both alleles coding for a serine amino acid.

### MicroRNA target detection

As previously mentioned, the polled concordant SNP bSYNJ1_C3981T is located in the 3'UTR of the gene *SYNJ1*, and 3'UTRs have been shown to be targets of a gene expression regulatory system orchestrated by microRNAs (*e.g*., microRNA regulation of myostatin gene expression impacts muscularity in sheep) [15]. In order to examine if the 3'UTR of *SYNJ1*, and specifically the location of SNP bSYNJ1_C3981T, may be a microRNA target, the current collection of bovine microRNA mature sequences (N = 117) was downloaded from the publicly available miRBase [16]. The microRNA sequences were used in target prediction analysis with the on-line resource RNAhybrid [17] using the parameters described (see Materials and Methods).

**Figure 2:**
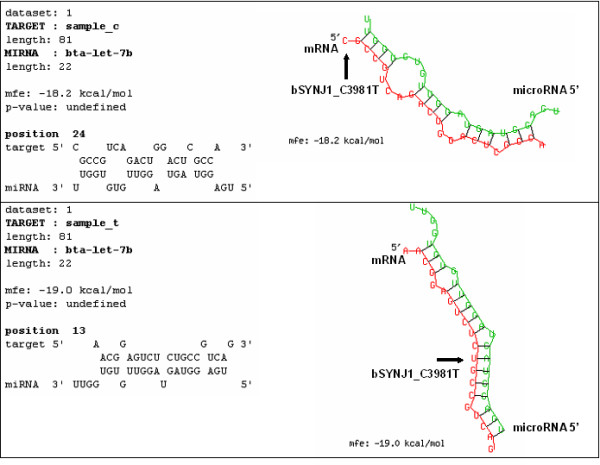
**RNAhybrid analysis of *SYNJ1 *3'UTR bSYNJ1_C3981T allelic variants (C allele = sample_c; T allele = sample_t) and bovine microRNA bta-let-7b**. The C allele does not align in the seed sequence of microRNA bta-let-7b (top pane), while the T allele does with one allowed gap (bottom pane).

Of the 117 bovine microRNAs currently available in miRBase [16], eight target the 3'UTR of *SYNJ1 *and overlap the location of SNP bSYNJ1_C3981T (Table 5). All bta-let-7 and the bta-mir-98 microRNA seed sequences (2–8 bp from the 5' end of the microRNA) [18] are disrupted with the bSYNJ1_C3981T allele concordant with the horn phenotype ('C'). All bta-let-7 and bta-mir-98 microRNA seed sequences appear functionally intact with the bSYNJ1_C3981T allele concordant with the polled phenotype ('T'). Disruption and alignment of the microRNA bta-let-7b seed sequence overlapping the location of SNP bSYNJ1_C3981T is shown in Figure 2. MicroRNA bta-mir-145 (Table 5) overlaps the location of the bSYNJ1_C3981T SNP, but the SNP is not within its seed sequence.

**Table 5:** MicroRNAs predicted to target the 3'UTR of *SYNJ1 *at the location of SNP bSYNJ1_C3981T.

**microRNA**	**miRBase Accession**	**Mature Sequence (5'-3')**	**Reference**
bta-let-7a	MIMAT0003844	UGAGGUAGUAGGUUGUAUAGUU	[29]

bta-let-7b	MIMAT0004331	UGAGGUAGUAGGUUGUGUGGUU	[29]

bta-let-7e	MIMAT0004333	UGAGGUAGGAGGUUGUAUAGU	[29]

bta-let-7f	MIMAT0003519	UGAGGUAGUAGAUUGUAUAGUU	[29]

bta-let-7g	MIMAT0003838	UGAGGUAGUAGUUUGUACAGUU	[29]

bta-let-7i	MIMAT0003851	UGAGGUAGUAGUUUGUGCUGUU	[29]

bta-miR-98	MIMAT0003809	UGAGGUAGUAAGUUGUAUUGUU	[30]

bta-miR-145	MIMAT0003542	GUCCAGUUUUCCCAGGAAUCCCU	[29]

## Discussion

A total of 13 novel SNPs concordant with the polled trait in Holsteins (Table 2) were identified. Because of the relatively small sample size and partial family structure (Figure 1) no association analysis was performed. These SNPs will require further testing in an unrelated set of Holsteins for verification. It is also not unexpected the Holstein breed is not validated for the commercial Tru-Polled™ test [6] as the polled trait has various historical origins in different breeds. One hypothesis is that when the causal functional DNA element (*i.e*., a gene or regulatory region) for polled is identified there may be multiple breed-specific mutations for the trait in that DNA element, if the trait arose separately in more than one breed. Another hypothesis is the trait was selectively introgressed from the first breed exhibiting polled and the causal mutation will be found to be the same in all breeds. The 13 SNPs reported here do represent the first described genetic markers for the polled trait specifically in Holsteins. In addition, these 13 SNPs are novel as they are not found in a search of public databases, such as NCBI's dbSNP [19] or the Bovine Genome Project's SNP database [13].

While discovery of polymorphisms concordant with the polled trait creates utility as a genetic test, the ultimate objective is characterization of a polymorphism with a probable functional effect for the polled trait. SNP bSYNJ1_C3981T is located in the 3'UTR of the *SYNJ1 *gene. The 3'UTR of genes has been shown to potentially be a target for microRNA regulation of gene expression by post-transcriptionally base-pairing to mRNAs [20]. *In silico *analysis of the *SYNJ1 *3'UTR allelic variant created by the bSYNJ1_C3981T SNP revealed eight predicted microRNA target interactions from the 117 available bovine microRNAs (Table 5). Over 500 human microRNAs have been characterized to date [16] indicating many more bovine microRNAs likely exist as well.

Six of the eight microRNAs (Table 5) are members of a microRNA family, bta-let-7. The let-7 microRNA family has been found to function in late development timing in *C. elegans*, and one of multiple targets of the let-7 microRNA in *C. elegans *is a nuclear hormone receptor *daf-12 *in the seam cells of the hypodermis [21]. The hypodermis is the lowermost layer of the integumentary system, and the integumentary system includes stratified squamous and keratinized epithelium. At a fundamental level, horns are epithelial with a bony core, living tissue, cornified, unbranched, permanent, cannot regenerate, and develop through basal growth [22].

Based on the *in silico *analysis presented in this report, it is possible to hypothesize the *SYNJ1 *3'UTR SNP (bSYNJ1_C3981T) alters a microRNA target site. The effect could be predicted to be tissue specific in such a manner that only horn growth is affected (as the mere presence or absence of horns alone has not been correlated with any other defect to the best of the authors' knowledge) by an unknown mechanism of *SYNJ1 *function. If the *SYNJ1 *3'UTR SNP does have a functional effect on the polled trait in Holsteins it, and the *SYNJ1 *gene itself, becomes a candidate for further investigations of potential causal mutations for the polled trait in other breeds.

## Conclusion

In summary, the polymorphisms reported here as concordant with the polled trait in Holsteins can readily be used as a genetic test for this breed. This is the first report, to the authors' knowledge, of SNPs within gene coding or regulatory regions (*i.e*., not introns or inter-genic) predictive of the horned/polled trait in cattle as previous reports localized and fine-mapped the polled region utilizing inter-genic genetic markers such as microsatellites [7-11]. These SNPs will require further testing for verification and further study to determine if the *SYNJ1 *3'UTR SNP may have a functional effect on the polled trait in Holsteins.

## Methods

### Animals and DNA samples

Twenty-four Holstein bulls were utilized as a polymorphism detection panel (Figure 1). The majority of the 24 Holsteins are directly related as horned sires and polled sons, with the dams expected to be polled (Figure 1). Semen samples from the bulls were purchased on the open market. In addition to de-horning, the scurs phenotype [2,23] may complicate identification of truly polled animals. Scurs was not investigated in this study as data was not available. The 12 polled Holstein bulls are registered as polled. All DNA samples were extracted from spermatozoa using the Qiagen Biosprint (Qiagen Inc., Valencia, CA) according to the manufacturer's protocol.

### PCR

All primers for PCR were designed using Primer3 [24]. Optimal primer annealing temperatures were obtained by using gradient PCR thermocycling conditions of 15 minutes at 95°C, 35 cycles of 45 seconds at 94°C, 45 seconds of gradient temperatures starting at 55° to 66° across twelve sample wells, 45 seconds at 72°C, and 10 minutes 72°C. Once an optimal annealing temperature was found, standard thermocycling conditions were used: 15 minutes at 95°C followed by 35 cycles of 45 seconds at 94°C, 45 seconds at optimal annealing temp, and 45 seconds at 72°C, with a final extension step of 10 minutes at 72°C. Concentrations for a 10 μl PCR volume (gradient and standard) were 5 ng/μl of template DNA, 0.5 μM of each primer (forward and reverse), 1× SIGMA JumpStart PCR Mix (Sigma-Aldrich Co., St. Louis, MO), and 1× combinatorial enhancer solution (CES) [25].

### Putative regulatory element prediction

The on-line resource WWW Promoter Scan [14] was used to scan targeted gene introns and inter-genic sequences for predicted regulatory elements such as promoters and transcription factor binding sites.

### Sequencing and analysis

PCR products were purified using the EXO-SAP-IT PCR Product Clean-up (USB corporation, Cleveland, OH) according to the manufacturer's protocol. Direct sequencing of purified PCR products, in both forward and reverse directions, was conducted using ABI BigDye (Applied Biosystems, Foster City, CA) according to the manufacturer's protocol and resolved on an ABI 3730xl Automated Sequencer (Applied Biosystems, Foster City, CA). Sequence trace alignment and polymorphism detection were carried out using recent versions of Phred/Phrap [26,27] and Consed [28].

### MicroRNA target prediction

The current collection of bovine microRNA mature sequences (N = 117) was downloaded from the publicly available miRBase [16]. The microRNA sequences were used in target prediction analysis with the on-line resource RNAhybrid [17]. Default target prediction parameters were used, specifically setting 1 hit per target and an energy cut-off of -14. A microRNA was considered to target the 3'UTR of a gene if the specified parameters were met and the seed sequence of the microRNA, base positions 2–8 from the 5' end of the microRNA [18], contained no more than one gap or mismatch with the 3'UTR sequence being tested.

## Competing interests

The authors declare a provisional patent application has been filed related to the content presented in this report and this work was fully funded by Monsanto Company.

## Authors' contributions

EJC participated in design of the study, contributed to selection of the Holstein panel, conducted all experimental analyses (*e.g*., polymorphism discovery, microRNA target detection), and drafted the manuscript. NJN participated in design of the study, contributed to selection of the Holstein panel, and provided review of the manuscript. MDG participated in design of the study, provided guidance for experimental analyses, and provided review of the manuscript.
